# Study of kidney morphologic and structural changes related to different ischemia times and types of clamping of the renal vascular pedicle

**DOI:** 10.1590/S1677-5538.IBJU.2018.0559

**Published:** 2019-09-02

**Authors:** Angela Mazzeo, Anna Paula Weinhardt Baptista Sincos, Katia Ramos Moreira Leite, Miguel Angelo Góes, Oscar Fernando Santos dos Pavão, Oskar Grau Kaufmann

**Affiliations:** 1 Hospital Israelita Albert Einstein, São Paulo, Brasil;; 2 Laboratório de Urologia, Faculdade de Medicina da Universidade São Paulo - USP, SP, Brasil;; 3 Disciplina de Nefrologia, Escola Paulista de Medicina, Universidade Federal de São Paulo - Unifesp, SP, Brasil

**Keywords:** Kidney, Ischemia, Nephrectomy

## Abstract

**Purpose:**

This study aimed to study morphological and renal structural changes in relation to different ischemic times and types of renal vascular pedicle clamping.

**Methods:**

Sixteen pigs were divided into two groups (n = 8): Group AV - unilateral clamping of the renal artery and vein and Group A - unilateral clamping of the renal artery only, both with the contralateral kidney used as control. Serial biopsies were performed at 0, 10, 20, 30, 40, 50, 60, 70, 80, and 90 minutes after clamping.

**Results:**

there is a correlation between the occurrence of renal damage as a function of time (p <0.001), with a higher frequency of Group A lesions for cellular alterations (vascular congestion and edema, interstitial inflammatory infiltrate, interstitial hemorrhage and cell degeneration), with the exception of in the formation of pigmented cylinders that were evidenced only in the AV Group.

**Conclusion:**

the number of lesions derived from ischemia is associated with the duration of the insult, there is a significant difference between the types of clamping, and the AV Group presented a lower frequency of injuries than Group A. The safety time found for Group A was 10 minutes and for Group AV 20 minutes.

## INTRODUCTION

Nephron-sparing renal surgery with renal ischemia (partial nephrectomy) is the gold standard technique, during which it is observed blood flow interruption to the vascular pedicle in order to reduce intra-operatory bleeding and to ease dissection. Kidneys, differently of other organs, endure lack of up to 20% of their blood flow. However, patients submitted to this procedure may develop acute transitory renal failure (that may last days, hours or even weeks). It also can cause definitive failure (1, 2).

There are two paradigms related to renal ischemia (RI). First, safety time to induce ischemia without harming renal function. Different values are reported, with a safety window varying from 25, 30 to 60 minutes. Some authors suggest a safety window of 25 minutes, while others report 30 or 60 minutes (3, 4). The second paradigm is associated to the type of clamping used to interrupt blood flow (5). Some authors recommend clamping only the renal artery, causing less harm to the kidney due to the presence of venous flow. Other studies suggest that clamping all renal vascular pedicle may result in lower number of renal lesions following surgery (6, 7).

Most studies using experimental models of ischemia-reperfusion collect samples before and after the surgical procedure (hours or even days) (8). Differently than the reference articles, the appointed study was made “in loco”, collecting renal parenchyma samples in timely intervals, in order to map histological alterations according to time (length) of renal ischemia. Pigs were used as experimental animals due to their anatomic and physiologic similarities to human kidney.

In the present study, we checked the main renal histological alterations according to different times of ischemia and types of clamping in pigs, in order to verify safety time for renal ischemia and significant differences between clamping techniques.

## MATERIAL AND METHODS

### Pigs

Large white female pigs two months old, weighting 25 to 30 kg, were used. This project was approved by the Ethical and Animal Experimentation Committee of the Hospital Israelita Albert Einstein, São Paulo, Brazil, protocol #2617.

### Study design

Sixteen pigs were used, divided in two major study groups: AV group (artery and vein) and A group (artery), distributed in 8/16 each: the right kidney was the control (no clamping) and the left kidney the experiment (clamped). Meaning that clamping was unilateral in both groups. Biopsy samples were collected timely at 0, 10, 20, 30, 40, 50, 60, 70, 80 and 90 minutes of clamping in each of the major study groups, bilaterally. Samples were kept in vials containing buffered formaldehyde 10% that were posteriorly processed and blocked in paraffin. Slices of 3μm were obtained and stained with HE technique (hematoxylin-eosin) and were analyzed at the optical microscope.

### Experimental protocol to induce ischemia

Animals were sedated via intramuscular with an injection of ketamine (10mg/kg body weight) and midazolam (0.25mg/kg body weight). After 15 minutes, a catheter caliber 20 or 22 (BD Insystem, BectonTherapy Systems Inc., USA) was inserted in the marginal vein of right ear to induce anesthesia with thiopental (7mg/kg body weight). Fastening was balanced with an initial dose of 2mL/kg/hour of rapid crystalloids and maintained with 10mL/kg/hour during surgical procedure. For endotracheal intubation it was used tubes 6.5 to 8.5 diameter (Portex®). Likewise, hypoxia was induced lowering oxygen inspired flow to 0.06-0.0, and anesthesia was maintained with inhaling isoflurane 2%. At the same time, current volume of 10mL/kg was maintained and analgesia was maintained with fentanyl (2-5mg/kg). Each animal was submitted to hemodynamics monitoring with invasive arterial pressure, heart rate and oxygen level. The anesthetized animal was kept in horizontal dorsal decubitus, local hygiene and with surgical fields with non-sterile technique. A midline abdominal incision was made to evaluate the kidneys with easy handling via retroperitoneal. Open surgical technique was chosen to access both kidneys (simultaneously) since laparoscopy does not allow for this procedure. Although bilateral, laparoscopy is not performed simultaneously (needing right and left lateral decubitus). With this approach, the right kidney (control) and the experimental (left kidney) would be approached simultaneously in each animal, lowering the number of animals needed. Next, left renal pedicle (AV group) and renal artery (A group) were clamped with a bulldog vascular clamp for 90 minutes, during which time the serial renal parenchyma biopsies, in the appointed times, were collected, using a scalpel #11. Biopsies were standardized in 1x1cm^2^ in the cortical region. After each biopsy, the local was compressed with gauze to control bleeding, that did not need any extra procedure for control (kidney pigs show a good hemostatic response). Serial biopsies did not affect negatively the kidneys, since they were performed at the cortex in different locals. After 90 minutes surgical clamps were removed, and in the end of the surgical procedure the animals were euthanized under general anesthesia with an overdose of thiopental and potassium chloride 19.1% IV (dose 15-30mg/kg).

### Experimental results

Ischemia effects were observed by histopathological qualitative analysis. The presence or absence of histological changes (frequency and prevalence) were compared between study groups according to time and type of clamping.

Described cellular alterations included: (a) degenerative alterations and vacuolization of tubular cells; (b) presence of pigmented cylinders; (c) vascular congestion; (d) edema; (e) interstitial neutrophilic infiltrate; and (f) interstitial hemorrhage.

### Statistical analysis

Results are presented in proportions, standard errors and 95% confidence intervals obtained by general estimative equation model (GEE) with binomial distribution.

Also, several multiple comparisons of medium values were made using the sequential Bonferroni test, using the SPSS software (IBM Corp. 2016). P <0.05 was considered statistically significant (9).

## RESULTS

In this study, we used a non-lethal model of renal lesion and no animal or data were excluded during analysis.

### Histological results

Forty biopsies were collected to analyze histologically each of the studied groups: Groups A and AV, n=10/20 kidney control and n=10/20, experimental kidney for each group.

**Figure 1:**
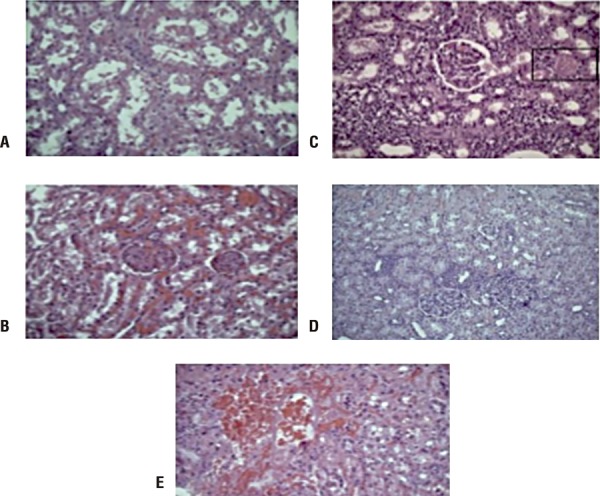
A) Tubular degenerative changes; B) pigmented cylinders; C) vascular congestion and edema; D) interstitial neutrophilic infiltrate; E) interstitial haemorrhage. All histological findings were stained with Hematoxylin-Eosin technique and analyzed under light microscopy.

Five tissue lesions were identified at renal biopsies: vascular congestion and edema, interstitial inflammatory infiltrate, degenerative alteration of tubular epithelial cells, pigmented cylinders and interstitial hemorrhage (Figure-1). However, in some instances, all biopsies in some of the groups showed determined lesion or any of them or in any group had any lesion. Therefore, it was not possible to evaluate the effects in the proposed times and groups using inferential methods or hypothesis tests.

Presence of vascular congestion and edema was observed in 90% of biopsies of ischemic kidney and 76.5% of control kidneys in Group-A. However, in AV Group, such lesions were present in 63.8% of ischemic kidney biopsies and 52.5% of control kidneys.

Tubular degenerative alterations (luminal dilatation, cytoplasm vacuolization, loss and peeling of epithelial cells) were observed mainly in proximal twisted tubules. The same alterations were identified in 67.5% of biopsies of ischemic kidneys and in 57.5% of control kidneys in Group A. In relation to AV group, the same was observed in 52.5% of biopsies of ischemic kidneys and in 43.7% of control kidneys.

Pigmented cylinders were observed in 6.25% of biopsies of ischemic kidneys and in none of control kidneys in Group A. In Group AV, the same lesion was seen in 30% of biopsies of ischemic kidneys and in 15% of control kidneys.

Interstitial hemorrhage was observed in 32.25% of biopsies of ischemic kidneys in Group A, and in 38.7% in control kidneys. In group AB, the lesion was observed in 5% of biopsies of ischemic kidneys and in 15% of control kidney biopsies.

Finally, the presence of interstitial inflammatory infiltrate was observed in 30% of biopsies of ischemic kidneys in group A and in 17.5% in control kidneys biopsies. The same alteration was found in relation to the number of cellular alterations in 15% of ischemic kidneys and 5% of control kidneys in Group AV.

**Figure 2:**
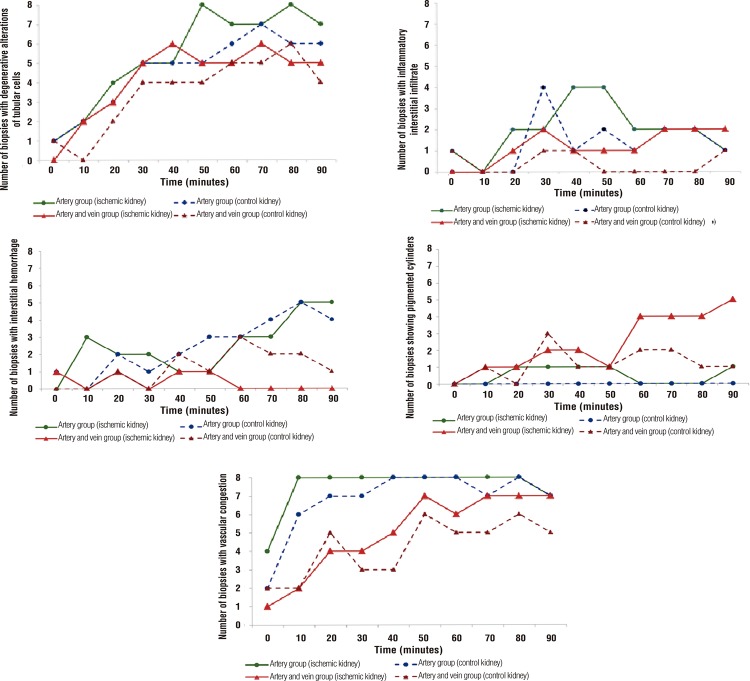
From left to right, the following cellular changes are observed as a function of the ischemia and type of clamping: degenerative tubular alteration, interstitial inflammatory infiltrate, interstitial hemorrhage, pigmented cylinders and vascular congestion.

The number of cellular alterations according to time and type of clamping is shown in Figure-2.

### Time (minutes)

- Artery group (ischemic kidney) Artery group (control kidney);
- Artery and vein group (ischemic kidney) Artery and vein group (control kidney);
- Number of biopsies with degenerative alterations of tubular cells;
- Number of biopsies with inflammatory interstitial infiltrate;
- Number of biopsies with interstitial hemorrhage;
- Number of biopsies showing pigmented cylinders;
- Number of biopsies with vascular congestion;

### Alterations according to clamping times

Analysis of total number of lesions was made using the General Estimative Equation model (GEE), that considered the total number of observed lesions in each biopsy (variable response), study groups (groups A and AV with respective ischemic and control kidneys-explicative variable) and clamping time (10, 20, 30, 40, 50, 60, 70, 80 and 90 minutes).

Table-1 shows the multiple comparisons among study groups (total number of lesions observed according to clamping time). After 20 minutes (p <0.001), there was a significative increase of the number of lesions, compared to basal time in ischemic kidneys in group A. Likewise, in the control kidneys, it was observed that at 30 minutes (p=0.001), 50 minutes (p <0.001), 70 minutes (p <0.001) and 80 minutes (p <0.001) there was significant increase of the number of lesions in comparison to basal time. On the other hand, in the ischemic kidneys of AV group there was significant increase of the number of lesions compared to basal time at 40 min (p <0.001), 50 min (p <0.001), 60 min (p=0.001), 70 min (p <0.001) and 90 min (p <0.001).

**Table 1:** Estimated proportions for the presence of lesion in the biopsies performed.

Comparison (min x Basal)	Study groups			

	Artery and vein		Artery	

	Control Kidney	Ischemic Kidney	Control kidney	Ischemic kidney
10	0.555	0.102	0.046	0.028
20	0.137	0.038	0.003	<0.001
30	0.135	0.004	0.001	<0.001
40	0.094	<0.001	0.003	<0.001
50	0.008	<0.001	<0.001	<0.001
60	0.001	0.001	0.003	<0.001
70	0.019	<0.001	<0.001	<0.001
80	0.001	0.002	<0.001	<0.001
90	0.137	<0.001	0.025	0.002

P values corrected by the sequential Bonferroni method

Finally, Table-2 shows the proportion of estimated lesions according to type of clamping; in Group A, after 20 minutes (both experimental and control kidneys) 20% an 32.5% showed a significant increase of the number of lesions according to the type of clamping, respectively. Also, in group AV, the ischemic kidneys showed after 20 minutes (25%) a significant increase of number of lesions. In kidney controls such increase was observed following 30 minutes (27.5%).

**Table 2:** Results of multiple comparisons between moments regarding the presence of lesions using Bonferroni Method with p value <0.001.

Basal (min)	Clamping type					

	Artery and vein		Artery			

	Control kidney n (%)	Ischemic kidney n (%)		Control kidney n (%)	Ischemic kidney n (%)	
0	10.0 (3.5)	7.5 (4.9)		10.0 (7.1)	17.5 (6.6)	
10	7.5 (3.4)	12.5 (4.9)		20.0 (3.5)*	32.5 (4.9)*	
20	20.0 (5.0)	25.0 (5.9)*		30.0 (6.1)*	42.5 (6.6)*	
30	27.5 (7.0)*	32.5 (4.9)*		42.5 (7.4)*	45.0 (5.9)*	
40	27.5 (6.1)*	37.5 (5.5)*		40.0 (5.0)*	47.5 (7.0)*	
50	30.0 (5.0)*	37.5 (6.6)*		45.0 (6.8)*	55.0 (5.9)*	
60	37.5 (8.2)*	40.0 (7.1)*		45.0 (6.8)*	50.0 (7.1)*	
70	35.0 (8.5)*	47.5 (7.0)*		50.0 (8.7)*	50.0 (5.0)*	
80	37.5 (5.5)*	45.0 (7.7)*		52.5 (7.9)*	57.5 (4.2)*	
90	30.0 (9.4)*	47.5 (7.0)*		45.0 (8.5)*	52.5 (8.6)*	

*Significant difference in relation to basal time. Data represent estimated proportions and standard errors obtained with the general estimative equation model.

## DISCUSSION

**Figure 3:**
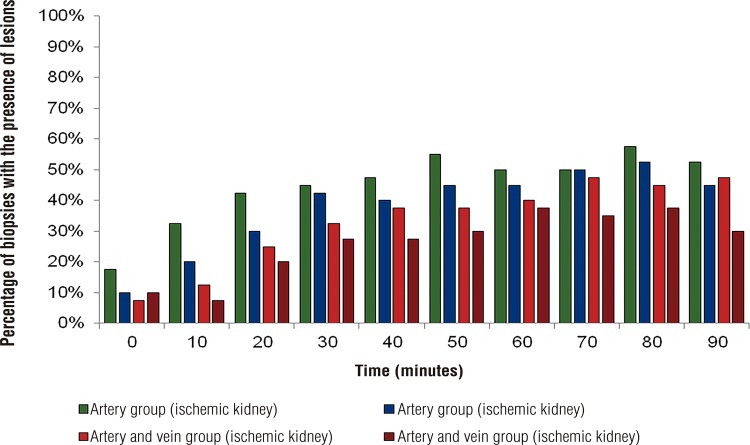
Distribution of the proportion of biopsies with presence of lesion.

When we analyze the occurrence of renal lesion only in relation to time, there is an interaction between these two variables (p <0.001): the number of lesions increases proportionally to the ischemia time (Figure-3). These results are coincident with those of literature (10, 11). Samples from ischemic kidneys show higher number of lesions compared to control kidneys.

### Percentage of biopsies with the presence of lesions

#### Time (minutes)

- Artery group (ischemic kidney)
- Artery group (control kidney)
- Artery and vein group (ischemic kidney)
- Artery and vein group (control kidney)

In relation to groups A and AV, most tissue lesions (vascular congestion and edema, tubular degenerative alterations, inflammatory interstitial infiltrate and interstitial hemorrhage) were more prevalent and frequent in Group-A in relation to group AV. Lesions were identified 10 minutes after induction of ischemia in Group-A and after 20 minutes in Group AV. The only alteration with higher prevalence and frequency in Group AV was the presence of pigmented cylinders, particularly in the ischemic kidney after 60 minutes. Based on these results, it was shown a significative difference between the types of clamping of renal pelvis. Early histological alterations were detected when only the renal artery was clamped, suggesting a renoprotective effect, compared to clamping renal artery and vein. These results are in accordance to Thompson et al., that demonstrated that renal artery clamping causes lower grade of renal alterations compared to clamping of renal artery and vein (12). However, our results are opposite of those of Mir et al., that suggested that clamping renal artery and vein during open surgery of pigs causes more harm them the clamping of only the renal artery, and that venous flow would benefit renal homeostasis (13). Chan et al. suggested that arterial clamping must be avoided since it causes vasospasm that are closely related to renal dysfunction (14).

In relation to the results of time, data of the AV group (20-30 minutes) are in accordance to those observed at literature, that recommends ischemia time between 25 and 30 minutes (Volpe et al., 5). In humans, there are several studies that considers that kidneys are able to endure 30 to 60 minutes of ischemia, without damaging renal function (15). It is known that renal lesion is to an extension reversible, and the identification of the transition time for irreversibility must still be determined. Variables such as surgical technique, patient’s age, comorbidities such as diabetes and hypertension, arterial vascularization and pre-operatory renal function determine that the ischemic damage must be reduced to a minimum, and it is suggested the use of hypothermia after 30 minutes of ischemia (16).

It is important to have in mind that factors such as the maintenance of anesthesia, monitoring blood pressure and hydration are fundamental for hemodynamics stability and reduction of negative effects on renal function (17).

The use of these data must guide the design of clinical trial protocols. In patients with normal pre-operatory function, 30 minutes is the safe considered time for ischemic injury. However, this value is been questioned by some researches that considered it a failed algorithm (18, 19). The quantity and quality of remaining renal parenchyma interfere directly in the recovery of renal function; however, in order to confirm this hypothesis, the use of new biomarkers, as well as more sensitive immune histochemical studies are required (20).

According to our study, the presence of lesion in control kidneys in both groups may be justified by crosstalk phenomenon (21). Crosstalk is observed when there is a lesion from a ischemia-perfusion event, that may diffuse to other organs such as lungs, liver and heart. Patho-physiologically, the lesion caused by ischemia-reperfusion promotes activation of pro-inflammatory and pro-apoptotic mediators, and the generation of oxidative stress and production of reactive oxygen species (ROS), that activate leukocytes and modify vasoactive cytokine levels (TNF-α, IL-1β, IL-6, IL-12, IL-15, IL-18, IL-32, and endotelin-1) and chemokines such as the product of leucocyte-endothelium adhesion and leucocyte activation (a characteristic process of ischemic lesion) (22).

The present study had some limitations, such as the reduced number of animals and the dichotomic qualitative statistical analysis that did not englobe all levels of renal lesion (mild, moderate and severe).

The safe time interval depends on several intrinsic and extrinsic variables of the surgery. However, literature shows a great number of studies that analyze differently the ischemic lesion. Until nowadays, there is no available algorithm to predict the risk of acute renal lesion in patients submitted to intra-operatory ischemia. Pre-operatory evaluation and planning surgical strategy in each case will bring better post-surgical results in relation to renal function maintenance.

## CONCLUSIONS

The number of cellular alterations caused by renal ischemia is associated to duration of ischemic injury. It was also verified that there is a significant difference between clamping only the renal artery and that AV group showed lower frequency of lesions than group A (that showed higher number of lesions in lower interval of ischemia time). Minimum time for the occurrence of histological lesions in group A was 10 minutes and for group AV was 20 minutes. The frequency of morphological and structural renal alterations are related to type of clamping and time of ischemia.
